# Vitamin D Receptor Gene Polymorphisms and Haplotypes in Hungarian Patients with Idiopathic Inflammatory Myopathy

**DOI:** 10.1155/2015/809895

**Published:** 2015-01-14

**Authors:** Levente Bodoki, Ji-Qing Chen, Margit Zeher, Melinda Nagy-Vincze, Zoltán Griger, Erika Zilahi, Katalin Dankó

**Affiliations:** Department of Clinical Immunology, Medical Faculty, University of Debrecen, Móricz Zs. Körút 22, Debrecen 4032, Hungary

## Abstract

Idiopathic inflammatory myopathies are autoimmune diseases characterized by symmetrical proximal muscle weakness. Our aim was to identify a correlation between VDR polymorphisms or haplotypes and myositis. We studied *VDR-BsmI*, *VDR-ApaI*, *VDR-TaqI*, and *VDR-FokI* polymorphisms and haplotypes in 89 Hungarian poly-/dermatomyositis patients (69 females) and 93 controls (52 females). We did not obtain any significant differences for *VDR-FokI*, *BsmI*, *ApaI*, and *TaqI* genotypes and allele frequencies between patients with myositis and healthy individuals. There was no association of VDR polymorphisms with clinical manifestations and laboratory profiles in myositis patients. Men with myositis had a significantly different distribution of *BB*, *Bb*, and *bb* genotypes than female patients, control male individuals, and the entire control group. Distribution of *TT*, *Tt*, and *tt* genotypes was significantly different in males than in females in patient group. According to four-marker haplotype prevalence, frequencies of sixteen possible haplotypes showed significant differences between patient and control groups. The three most frequent haplotypes in patients were the *fbAt*, *FBaT*, and *fbAT*. Our findings may reveal that there is a significant association: *Bb* and *Tt* genotypes can be associated with myositis in the Hungarian population we studied. We underline the importance of our result in the estimated prevalence of four-marker haplotypes.

## 1. Introduction

Idiopathic inflammatory myopathies (IIMs) are autoimmune diseases characterised by proximal symmetrical muscle weakness. Adult polymyositis (PM)/dermatomyositis (DM), juvenile polymyositis (JPM)/dermatomyositis (JDM), overlap myositis (OM), inclusion body myositis (IBM), and necrotizing autoimmune myopathy (NAM) all belong to IIM. According to one of the biggest analyses, in which forty-six articles—published between 1966 and 2013—were searched, the incidence of IIMs ranges from 1.16 to 19/million/year and the prevalence ranges from 2.4 to 33.8/100000 inhabitants [1]. In this study authors found disparities in the male-female ratio, according to geographical origin [1]. Our workgroup previously found that the male-female ratio among three hundred and thirty-seven Hungarian IIM patients was 1 : 2.92 [2]. Ethnical differences and HLA-associations suggest that genetic factors also may play a part in the aetiology.

Vitamin D is essential for normal development and maintenance of bones. It also impacts immunoregulation, enhances the production of interleukin- (IL-) 1, and obstructs the production of IL-2 [3, 4]. Vitamin D is considered as a regulator of the immune system [5, 6]. Most biological activities of vitamin D are mediated by the vitamin D receptor (VDR), which is located in the nucleus [7]. VDR acts as a transcription factor activated by a ligand and is encoded by the vitamin D receptor gene [8]. The VDR gene is located on* chromosome 12* (*12q12*-*q14*) [9]. In recent years many polymorphisms have been identified in the VDR gene, but their effects on VDR activity are still poorly understood [10]. The VDR gene has more than 100 restriction endonuclease recognition sites, but only 4 of them are known polymorphisms:* FokI*,* BsmI*,* ApaI*, and* TaqI*.* FokI* is located in exon II, while* BsmI* and* ApaI* (in intron 8) and* TaqI* (in exon 9) have been identified at the 3′ end of the gene. The effects of vitamin D and the VDR gene polymorphisms are in connection with each other. Nowadays, there is evidence that these polymorphisms have a relationship to such diseases or conditions where vitamin D has an essential role, for example, bone mineral density, osteoporosis and cancers of the breast and prostate [11–13]. According to Ferrari et al. VDR gene allelic variants predict bone mineral density and the effect of calcium intake on maintenance of bone mass could relate to VDR gene polymorphisms [11]. A study from Iran lends support to an increased risk of breast cancer associated with the* VDR*-*BsmI* polymorphism [12]; one Chinese study says that VDR gene polymorphisms may be potential risk factors for prostate cancer [13].

The latest findings suggest that allelic variations of VDR gene may partially represent a genetic component associated with incidence, clinical symptoms, or severity of autoimmune diseases or to the susceptibility of them [14–22]. It is notable that none of these studies shows a causative relationship between genes and disease development.

To the best of our knowledge, there is no information in the literature about the VDR gene polymorphisms and their association with myositis disease. Therefore the aim of this study was to determine whether an association exists between VDR gene polymorphisms and IIM. We assessed the VDR gene* BsmI*,* ApaI*,* TaqI*, and* FokI* polymorphisms in patients with IIM and healthy controls to assess whether a relationship exists between polymorphisms/haplotypes in the VDR gene and susceptibility to PM/DM. Our aim was also to identify whether an association exists between VDR gene polymorphisms and some significant markers of myositis: the phenotype of disease, age at disease onset, gender, the presence of myositis-specific autoantibodies, or the presence of osteoporosis.* BsmI*,* ApaI*, and* TaqI* have been shown to be in strong linkage disequilibrium (LD) [23]. LD means that there is an association of alleles of adjacent polymorphisms with each other. LD in combination with one or more functional polymorphisms elsewhere in VDR gene is believed to explain the observed associations between the VDR gene and diseases. Another aim of the study was to establish haplotype analysis, which has recently become important due to newly developed field of bioinformatics.

## 2. Methods

### 2.1. Patients and Controls

89 patients with IIM were enrolled in the present study, recruited from the Autoimmune Outpatient Clinic of the Department of Clinical Immunology, Medical Faculty, University of Debrecen. The patient group included 69 female and 20 male patients; male-female ratio was 1 : 3.45; this is similar to our previous results [2]. The mean age was 52.6, ranging from 9 to 81 years. One limitation of the study is that the patient cohort was very heterogeneous because of diseases with different pathogenesis. The distribution of IIM patients was as follows: PM, *n* = 46; DM, *n* = 15; JPM/JDM, *n* = 9; OM, *n* = 17 (myositis and RA in 10 patients; myositis and progressive systemic sclerosis (PSS) in 5 patients; myositis and mixed connective tissue disease (MCTD) in 2 patients); NAM, *n* = 1; IBM, *n* = 1. The diagnosis of PM and DM was established according to the criteria determined by Bohan and Peter [24]: (1) symmetrical proximal muscle weakness, (2) positive muscle biopsy, (3) elevation of serum skeletal muscle enzymes, (4) myopathic triad on electromyography, and (5) characteristic skin symptoms (Gottron's signs, Gottron's papules, V-sign, scarf sign, erythematous rashes, heliotrope rash, periungual capillary changes, and periorbital oedema) in DM. The diagnosis of JPM and JDM was established when the first symptoms of myositis developed under the age of eighteen. In the case of OM myositis occurred together with other connective tissue diseases (CTDs) such as RA, PSS, or MCTD. It has to be laid down that the Bohan and Peter criteria are mainly employed to refer to PM and DM. That is why the Hilton-Jones MRC criteria have to be used for IBM which is a late onset myopathy with both inflammatory and myodegenerative features [25]. NAM patients present with a subacute severe symmetrical proximal myopathy, associated with a markedly elevated creatine kinase level. NAM patients must have the common histopathological features of myocyte necrosis without significant inflammation [26].

A total of 93 healthy individuals (mean age 41.2 years, range 14 to 70 years, 52 females and 41 males) taking no immunosuppressive or immunomodulating medications served as the control group.

Informed consent was obtained from all of the subjects. All experiments carried out were in compliance with the Declaration of Helsinki.

### 2.2. Detection of Myositis-Specific Autoantibodies (Anti-Jo-1, Anti-PL-7, Anti-PL-12, Anti-Mi-2, and Anti-SRP)

These antibodies were detected by membrane-fixed immunoblot (Orgentec Diagnostika). The method is briefly described. First, the contents of each vial of buffered wash solution concentrate were diluted with distilled water to a final volume of 1000 mL prior to use. A myositis plus strip was inserted and 1 mL of sample buffer was added to each chamber of the incubation tray. Then, it was equilibrated for 5 min with gentle rocking. 10 *μ*L of sera was added to each chamber and incubated for 60 min at room temperature. Then, the diluted serum was removed from the strips. 2 mL of wash buffer was added; it was incubated for 5 min and then removed. This procedure had to be repeated twice. 1.0 mL of the enzyme conjugate was added to each chamber and incubated for 30 min. After removing the diluted conjugate, 2.0 mL of wash buffer was added and it was incubated for 5 min; then, it was removed and this step was repeated further two times. After adding 1 mL of substrate to each strip, a 10 min incubation was performed and, after removing the substrate, it was washed with 1 mL of distilled water, three times [27].

### 2.3. Detection Myositis-Associated Antibodies

Enzyme-linked immunosorbent assay (ELISA) was used for the detection of the following myositis-associated antibodies: rheumatoid factor (RF), anticyclic citrullinated peptide (anti-CCP), and anti-dsDNA (anti-double-strand deoxyribonucleic acid).

Anti-Ku, anti-PM-Scl, anti-U1RNP, anti-SSA, and anti-SSB were detected with immunoblot.

### 2.4. Detection of Extramuscular Manifestations in Myositis Patients

The presence of cardiac involvement (myocarditis and pericarditis) was investigated by echocardiography. The gastrointestinal disease activity was checked by asking the patient about the presence of dysphagia. Every single myositis patient's thorax was controlled by high resolution computer tomography and the diffusing capacity of the lung for carbon monoxide (DLCO). These are useful in judgment of the presence of interstitial lung disease. Arthritis was detected by physical examination and by X-ray.

### 2.5. Genomic DNA Extraction

Genomic DNA for genotyping was extracted from peripheral blood, which was collected in EDTA vacutainers. Genomic DNA was extracted according to the manufacturer's recommendation using a QiaAmp DNA Blood Mini Kit (Qiagen GmbH, Germany).

### 2.6. DNA Quantification

DNA was quantitated by UV absorption at 260 nm and 280 nm and stored at −20°C until analysed.

### 2.7. Genotyping of the* BsmI* Polymorphism (rs1544410)

Genotyping of the* BsmI* polymorphism was carried out in PCR-amplified DNA by allelic discrimination using TaqMan from Applied Biosystems (Foster City, CA, USA). PCR primers and TaqMan probes specific for the* BsmI* polymorphism were purchased from Applied Biosystems. The assay enables scoring of both alleles in a single well. Real-time PCR was performed using Corbett Rotor-Gene RG-3000 equipment.

The PCR reaction was carried out in a 20 *μ*L reaction volume containing TaqMan Universal Master Mix (2x, Applied Biosystems), TaqMan genotyping assay (40x), and optimised quantities of genomic DNA. The Universal Master Mix contained AmpliTaq Gold DNA polymerase, AmpErase UNG, dNTPs with dUTP, passive reference, and optimised buffer components. Reactions were set up in duplicate. Thermal cycling was initiated by incubation at 95°C for 10 min for optimal AmpErase UNG activity and the activation of Amplitaq Gold DNA polymerase. After this initial step, 40 cycles of PCR were performed; each PCR cycle consisted of heating to 92°C for 15 sec for denaturation of the DNA and then cooling to 60°C for 1 min for annealing and extension.

### 2.8. Genotyping of* FokI*,* ApaI*, and* TaqI* Polymorphisms

The genotypes of the VDR gene* FokI*,* ApaI*, and* TaqI* polymorphisms were determined according to the digestion pattern generated for the amplified DNA fragment using the restriction enzymes* FokI*,* ApaI*, and* TaqI*.

#### 2.8.1. *FokI* Polymorphism (rs2228570)

Genotypes for the* FokI* polymorphisms were studied by PCR using the primers described in Table 1 [28]. PCR products were amplified in a programmable thermal cycler (Eppendorf-MC-EP model). The PCR conditions used to amplify the* FokI* SNP are summarised in Table 1. The amplified products were digested by incubation with the* FokI* enzyme (Fermentas Life Sciences) for 1 hour at 37°C, according to the manufacturer's instructions; products were electrophoresed on a 3% agarose gel and visualised by SYBR Green I staining.* FokI* genotypes were defined by capital letters in the absence of the restriction site (allele-*F*) and lowercase letters where the restriction site was present (allele-*f*).

#### 2.8.2. *ApaI* and* TaqI* Polymorphisms (rs7975232 and rs731236)

Genotypes for the* ApaI* and* TaqI* polymorphisms were studied by PCR using the primers as described in Table 1 [29]. The PCR conditions to amplify the* ApaI* and* FokI* SNPs are summarised in Table 1. The PCR products were digested by incubation with* ApaI* (Fermentas Life Sciences) for 4 hours at 37°C and* TaqI* (Fermentas Life Science) for 1 hour at 60°C. After digestion, the fragments were separated by electrophoresis in 3% agarose gels and visualised by SYBR Green I staining. For both* ApaI* and* TaqI*, genotypes were defined by capital letters in the absence of the restriction site (*A* and* T*, resp.) and lowercase letters where the restriction site was present (*a* and *t*, resp.).

### 2.9. Statistical Analysis

Genotype frequencies were calculated by direct counting. Allele frequencies were calculated from genotype frequencies based upon Hardy-Weinberg equilibrium. For comparisons of mean values between patients and controls, statistical analysis was performed using the independent samples *t*-test. Differences in genotypic and allelic distribution of VDR polymorphisms between patients and controls were determined by Pearson Chi-square (*χ* ^2^) test using SPSS 20.0 statistical software. The *P* value less than .05 was regarded as statistically significant. Haplotype analysis was performed by CHAPLIN 1.2 software. Pairwise linkage disequilibrium (LD) between the VDR gene polymorphisms was computed, and LD plots were constructed using Haploview software, version 4.2 [30].

## 3. Results

We studied* VDR*-*BsmI*,* VDR*-*ApaI*,* VDR*-*TaqI*, and* VDR*-*FokI* polymorphisms in 89 polymyositis/dermatomyositis patients and 93 healthy controls.

### 3.1. Allele Frequencies of VDR Gene Polymorphisms in IIM Patients

The distribution of allelic frequencies for the four polymorphisms studied here is summarised in Table 2. The characteristics are shown separately for three main phenotype groups: (1) PM (including one patient with JPM, one patient with IBM, and one patient with NAM); (2) DM (including 8 patients with JDM); and (3) overlap patients. No significant difference was found for allele frequencies when data were compared between patients with IIM and control individuals. We identified a difference in the frequency of* F* and* f* alleles between the IIM population and the control population, but this was not significant. In addition, no significant difference was observed when the IIM cases were grouped into PM, DM, and overlap cases.

### 3.2. Genotype Frequencies of VDR Gene Polymorphisms in IIM Patients

No significant difference was found in the genotype frequencies when the VDR gene genotypes of patients with IIM and healthy individuals were compared (Table 2). The same result was confirmed between PM, DM, and overlap cases (Table 2). However, we detected slightly increased prevalence of the* AA* genotype in IIM patients compared with controls: 37.07% versus 24.73%, respectively. The frequency of* Aa* genotype was slightly decreased in IIM patients compared with the control population: 39.33% and 50.54%, respectively. Some other notable differences were as follows: (a) the* TT* genotype was more frequent in PM patients than in DM patients (51.02% versus 34.78%, resp.); (b) the* Tt* genotype was less frequent in patients with PM than patients with DM (28.57% versus 52.18%, resp.); (c) the frequency of the* FF* genotype was lower in IIM patients than in controls: 26.97% versus 42.25%, respectively.

The patients were classified into groups according to their gender, age at disease onset, laboratory profiles (myositis-specific and myositis-associated autoantibody positivity), and the presence of osteoporosis. The most important clinical manifestations that are of great importance to the prognosis of IIM were also investigated. These are the following extramuscular manifestations: the presence of cardiac involvement (myocarditis), esophagus involvement (dysphagia), other skeletal involvements (arthralgia, arthritis), and lung involvement (interstitial lung disease). There was no association of VDR gene polymorphisms with clinical manifestations in IIM patients.

We detected important associations when investigating gender. The statistical *t*-test showed significant differences as follows: the distribution of* BB*,* Bb*, and* bb* genotypes was found to be significantly different (*P* < .001) in males compared to females among myositis patients (Table 3). We also found a similar distribution for* VDR*-*TaqI* polymorphism. The distribution of* TT*,* Tt*, and* tt* genotypes was significantly different (*P* = .037) in male myositis patients than in female myositis patients, as shown in Table 3. We also found statistically significant differences as follows: (a) comparing the male myositis patients with the entire control group, we established that the distribution of* BB*,* Bb*, and* bb* genotypes was significantly different (*P* = .0323) in the male IIM patients, (b) the distribution of the* BB*-*Bb*-*bb* genotype in the male patient group versus the men in the control group also showed a significant difference (*P* = .0176), (c) significant difference was detectable when comparing the frequency of* FF*,* Ff*, and* ff* genotypes between myositis-associated autoantibody positive patients and the control population (*P* = .0033), and (d) when examining the distribution of* AA*,* Aa*, and* aa* genotypes in female myositis patients and control female patients, the *P* value was .0398 (Table 3). Finally, comparing the distribution of* AA*,* Aa*, and* aa* genotypes between myositis patients with other autoimmune disease (overlap cases) and controls the *P* value was found to be .054.

### 3.3. Linkage Disequilibrium and Haplotype Frequencies of VDR Gene Polymorphisms in IIM Patients

**Figure 1:**
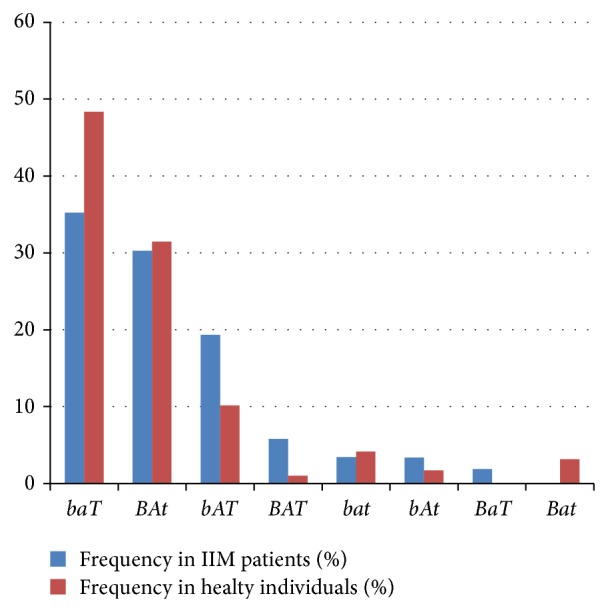
Three-marker (*BsmI*,* ApaI*, and* TaqI* polymorphisms) haplotype estimated prevalence (%) in IIM patients and healthy individuals (IIM: idiopathic inflammatory myopathy).

The haplotypes might provide valuable data where genotypes alone are not often able. The calculated haplotype frequencies for* VDR*-*BsmI*,* VDR*-*ApaI*, and* VDR*-*TaqI* polymorphisms of the IIM patients and controls are shown in Figure 1. There was no significant difference in the calculated haplotype frequencies between the control and the patient groups. The* baT* and the* BAt* haplotypes were the most frequent in both the patient group (35.22% and 30.28%, resp.) and the control population (48.35% and 31.46%, resp.).* bAT* haplotype was not as frequent as the above-mentioned haplotypes but was also frequent in both groups (19.33% in patients versus 10.16% in healthy population). The combined frequency of the five remaining haplotypes was less than 10%. The* Bat* was not present in the patient group, and in the control group, the estimated frequency of* BaT* was 0%.

**Figure 2:**
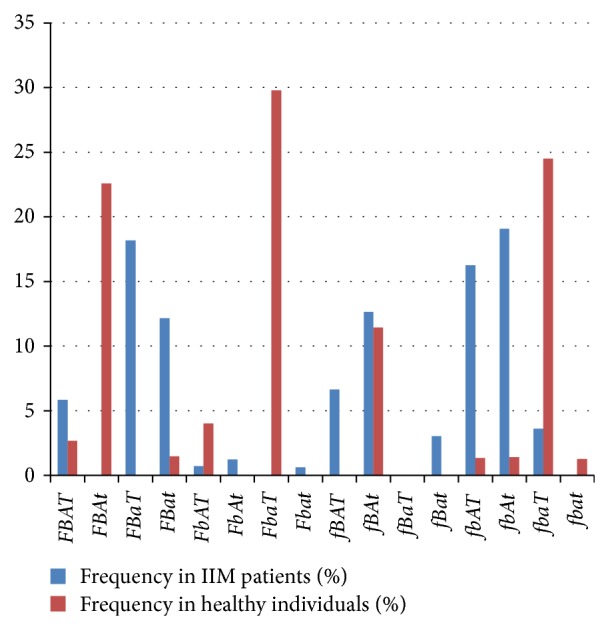
Four-marker (*FokI*;* BsmI*;* ApaI*; and* TaqI* polymorphisms) haplotype estimated prevalence (%) in IIM patients and healthy individuals (IIM: idiopathic inflammatory myopathy).

The distribution of the frequency of four-marker haplotype alleles (*VDR*-*FokI*,* VDR*-*BsmI*,* VDR*-*ApaI*, and* VDR*-*TaqI*) in IIM cases and controls is shown in Figure 2. According to the diagram, there are significant variances in the distribution. The most frequent haplotype in IIM patients was the* fbAt* haplotype (19.07%); the estimated prevalence of this haplotype in controls was only 1.41%. The second and third most frequent ones in patients were* FBaT* and* fbAT* (18.18% and 16.25%, resp.), while the estimated prevalence in the control group was only 0% and 1.34%, respectively. We should highlight here that, according to our findings, the three most frequent four-marker haplotypes in healthy individuals were as follows:* FbaT* (29.78%),* fbaT* (24.50%), and* FBAt* (22.59%). The estimated frequency of these in patients was 0%, 3.61%, and 0%, respectively.

**Figure 3:**
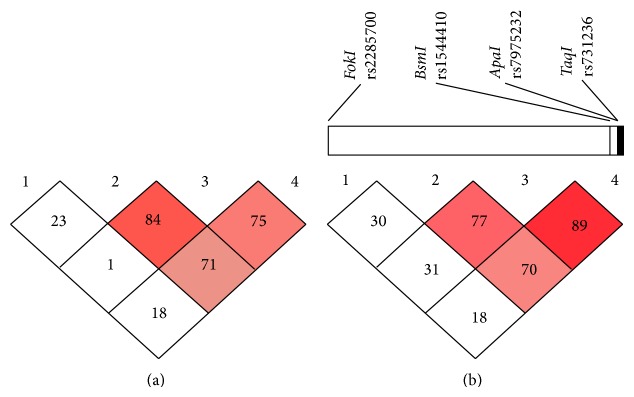
Four common gene polymorphisms of linkage disequilibrium of VDR gene of IIM patients (a) and the healthy controls (b). Graphical presentation of the VDR gene with the location of polymorphisms studied. Numbers in the boxes represent the correlation coefficient value of linkage disequilibrium (*r* ^2^) value multiplied by 100. The intensity of the dark color of the boxes represents strength of linkage disequilibrium (*r* ^2^) with dark boxes having high LD and white boxes having low LD. (LD: linkage disequilibrium).

LD analysis revealed a very strong LD (*r* ^2^ > .8) between* ApaI* and* TaqI* polymorphisms, a strong LD (*r* ^2^ between .67 and .7) between* BsmI* and* ApaI* or* BsmI* and* TaqI* polymorphisms, and a very weak LD (*r* ^2^ < .3) between* FokI* and other polymorphisms in the control group. In patients, very strong LD was found between* BsmI* and* ApaI* polymorphisms; moderate LD was found between* TaqI*-*ApaI* and* TaqI*-*BsmI* polymorphisms. No LD was observed between* FokI* and other polymorphisms in patients (Figure 3).

## 4. Discussion

VDR gene polymorphisms have been identified and analyzed so far in a wide variety of diseases, including Parkinson disease, cancer of the breast, and autoimmune diseases [23, 31, 32]. There are many workgroups from the Far East that investigated this field. One Chinese study found that there was an association between* Aabb* genotypes and the incidence of systemic lupus erythematosus (SLE);* Aabb* was involved in some clinical conditions and in antibody-production [14]. Results of another Chinese study indicated a possible role of the* B* allele in influencing SLE susceptibility [15]. By multiple sclerosis (MS) the results are controversial. Some articles [16, 17] neglect any association of VDR gene polymorphisms with disease susceptibility; however a Russian article refers to different clinical manifestations [18]. VDR polymorphisms may contribute to the high prevalence of RA in North American natives [19]. A Polish study said that* BsmI* may show some correlation with RA activity and progression [20]. According to an article from Spain* bb* genotype is associated with less severe disease [21]. There is some information about the VDR polymorphisms and the autoimmune thyroid diseases (AITD), as well [22]. One limitation of the mentioned studies is that they were carried out in different countries, preeminently in the Far East; they are distinct populations and the comparison with Central European data is not suitable.

The Central European literature about the possible association of VDR gene polymorphisms and autoimmune diseases is very limited. To the best of our knowledge there is no information in the literature about VDR gene polymorphisms and their connection with myositis. In our study, we determined VDR gene* BsmI*,* ApaI*,* TaqI*, and* FokI* polymorphisms in patients with IIM and healthy controls to analyse whether a relationship exists between the polymorphisms/haplotypes in VDR gene and susceptibility to PM/DM. Our most important findings were connected to the* VDR*-*BsmI* polymorphism,* VDR*-*TaqI* polymorphism, and male gender. Based on our data, we can state that the distribution of* BB*,* Bb*, and* bb* genotypes represents an interesting finding because* BB* and* bb* genotypes were presented at a very low percentage in the male patient population. The distribution of* TT*,* Tt*, and* tt* genotypes was significantly different in males than in females in the patient group. Our findings may reveal that IIM can be associated with the* Bb* genotype of the* VDR*-*BsmI* polymorphism and the* Tt* genotype of the* VDR*-*TaqI* polymorphism in males in our cohort.

As mentioned before, Hitchon et al. [19] found that RA is associated with the* FokI* polymorphism of the VDR gene. In our case-control study, 17 patients were included with OM, and 10 of these had RA. Comparing the distribution of* FF*,* Ff*, and* ff* genotypes of the VDR gene identified a significant difference between myositis-associated autoantibody positive patients and the control population. The* Ff* genotype was found to be the most frequent genotype in myositis-associated antibody positive cases (66.67% out of the positive patients). Also, 42.86% of the MAA patients were RF-positive, which confirms the results reported by Hitchon et al. [19].

The* baT* and* BAt* haplotypes were found to be the most frequent three-marker haplotypes in both the patient and control groups. These three-marker haplotypes were identified as the most frequent and corresponded to haplotypes reported by Morrison et al. [33].

Our data demonstrated that the frequencies of the sixteen possible haplotypes showed significant differences between the patient and the control groups when the four-marker haplotype prevalence was assessed. Several differences that were not statistically significant might accumulate and recombine in individuals resulting in significant differences in the level of four-marker haplotypes. This is the reason why certain haplotypes are represented in patients but are not found in control individuals according to the statistical calculations (Table 2 and Figure 2).

## 5. Conclusions

This is the first study to investigate the four known polymorphisms (*FokI*,* BsmI*,* ApaI*, and* TaqI* polymorphisms) in the VDR gene in patients with IIM. We found that a relationship may exist between the* BsmI* genotype and IIM in males and* TaqI* genotype and IIM in males as well. The most important limitation of the study is that our cohort was small. Males' number is limited to twenty, so we cannot claim certain assessments about the difference in polymorphisms prevalence in males and women.

In our study, we identified an important difference in the estimated prevalence (%) of the four-marker (*FokI*;* BsmI*;* ApaI*; and* TaqI* polymorphisms) haplotypes between IIM patients and healthy individuals. The three most frequent haplotypes in patients were the* fbAt*,* FBaT*, and* fbAT*, while this ranking in the control group was as follows:* FbaT*,* fbaT*, and* FBAt*. The pathomechanism of IIM is still unclear, but the genetic predisposition and environmental conditions play an important role. According to our findings, the genotype variations of the VDR gene polymorphisms may be one of the factors in the development of IIM which is a polygenic and multifactorial autoimmune disease.

## Figures and Tables

**Table 1:** Sequences of specific primers and the profile of PCR used to amplify the DNA fragment carrying *FokI*, *ApaI*, and *TaqI* SNPs [28, 29].

	*FokI* polymorphism (rs2228570)	*ApaI* and *TaqI* polymorphisms (rs7975232 and rs731236)
Primers	5′-AGC TGG CCC TGG CAC TGA CTC TGC TCT-3′	5′-CAG AGC ATG GAC AGG GAG CAA G-3′
	5′-ATG GAA ACA CCT TGC TTC TTC TCC CTC-3′	5′-GCA ACT CCT CAT GGC TGA GGT CTC A-3′

PCR conditions (40 cycles)	30 sec at 95°C	30 sec at 94°C
	30 sec at 70°C	1 min at 70°C
	30 sec at 72°C	1 min at 72°C

Length of PCR product (bp)	265	740

**Table 2:** Distribution of *VDR-FokI*, *VDR-BsmI*, *VDR-TaqI*, and *VDR-ApaI* genotypes in our Hungarian cases and controls.

Enzyme analysis	IIM patients (89 patients)	PM, JPM, IBM, NAM (49 patients)	DM, JDM (23 patients)	Overlap (17 patients)	Controls (93 person)
*BsmI polymorphism *					
Genotypes					
* BB *	19,1%	18,37%	8,69%	35,3%	15,9%
* Bb *	39,33%	36,73%	47,83%	35,3%	46,2%
* bb *	41,57%	44,9%	43,48%	29,4%	37,8%
Allele frequencies					
* B *	0,387	0,367	0,326	0,529	0,39
* b *	0,612	0,633	0,674	0,471	0,61

*ApaI polymorphism *					
Genotypes					
* AA *	37,07%	34,69%	30,43%	52,94%	24,73%
* Aa *	39,33%	38,78%	52,17%	23,53%	50,54%
* aa *	23,6%	26,53%	17,4%	23,53%	24,73%
Allele frequencies					
* A *	0,567	0,541	0,565	0,647	0,5
* a *	0,433	0,459	0,435	0,353	0,5

*TaqI polymorphism *					
Genotypes					
* TT *	43,82%	51,02%	34,78%	35,3%	40,86%
* Tt *	37,08%	28,57%	52,18%	41,17%	39,79%
* tt *	19,1%	20,41%	13,04%	23,53%	19,35%
Allele frequencies					
* T *	0,623	0,653	0,609	0,559	0,61
* t *	0,376	0,347	0,391	0,441	0,39

*FokI polymorphism *					
Genotypes					
* FF *	26,97%	30,61%	21,74%	23,53%	42,25%
* Ff *	48,31%	42,86%	52,17%	58,82%	43,66%
* ff *	24,72%	26,53%	26,09%	17,65%	14,08%
Allele frequencies					
* F *	0,511	0,52	0,478	0,529	0,64
* f *	0,489	0,48	0,522	0,471	0,36

**Table 3:** The distribution of various genotypes in our Hungarian myositis patients and healthy individuals (representing the cases with a *P* value less than .05).

	*BB *	*Bb *	*bb *	
Number of male myositis patients	1	15	4	*P* value < .001
Number of female myositis patients	16	20	33	

	*BB *	*Bb *	*bb *	

Number of male myositis patients	1	15	4	*P* value = .0323
Number of control individuals	16	40	37	

	*BB *	*Bb *	*bb *	

Number of male myositis patients	1	15	4	*P* value = .0176
Number of control male individuals	8	15	18	

	*TT *	*Tt *	*tt *	

Number of male myositis patients	5	12	3	*P* value = .037
Number of female myositis patients	34	20	15	

	*FF *	*Ff *	*ff *	

Number of MAA positive patients	2	14	5	*P* value = .0033
Number of control individuals	31	33	29	

	*AA *	*Aa *	*aa *	

Number of female myositis patients	28	23	18	*P* value = .0398
Number of control female individuals	11	28	13	
